# Increasing literate and illiterate women’s met need for contraception via empowerment: a quasi-experiment in rural India

**DOI:** 10.1186/1742-4755-11-74

**Published:** 2014-10-21

**Authors:** Federico R León, Rebecka Lundgren, Irit Sinai, Ragini Sinha, Victoria Jennings

**Affiliations:** León & Bustamante Consultores, Lima, Peru; Institute for Reproductive Health, Georgetown University, Washington, DC USA; Futures Group, Washington, DC USA; Xavier Institute of Social Service, Ranchi, Jharkhand India

**Keywords:** Women’s empowerment, Literacy, Met need for contraception, Intervention

## Abstract

**Background:**

Virtually all the evidence on the relationship between women’s empowerment and use of contraception comes from cross-sectional studies that have emphasized macrosocial factors.

This analysis tested whether literate and illiterate women are empowered by an intervention designed to provide information addressing technical and gender concerns and expand contraceptive choice, and evaluated the effects of women’s decision-making power on contraceptive behavior.

**Methods:**

The data came from a three-year quasi-experiment conducted in two comparable, yet not equivalent, rural blocks in Jharkhand, India. At the intervention block, a new contraceptive method was introduced at Ministry of Health health centers, providers were trained to offer family planning information and services which took into consideration gender power dynamics, and promotional messages and information about contraception were disseminated community-wide. Married women ages 15–49 who lived in the intervention and control blocks were sampled and interviewed before and after the intervention by a professional research firm. Data analyses included generalized linear models with interactions and covariate control.

**Results:**

Women’s normative beliefs concerning wives’ power in decisions regarding money earned and visits to relatives and friends vis-à-vis their husbands’ power were increased by the intervention; similar was the case among illiterate, but not literate, women regarding decisions related to childbearing. Concerning met need for contraception, the change for women with relatively more power who were illiterate was greater in the intervention than in the control area.

**Conclusion:**

The findings suggest that women were empowered by outreach visits that addressed gender dynamics and that their empowerment contributed to their met need for contraception. Generalizations to other settings, however, may be limited by cultural differences.

**Electronic supplementary material:**

The online version of this article (doi:10.1186/1742-4755-11-74) contains supplementary material, which is available to authorized users.

## Background

About 17 percent of all married women in the world would prefer to avoid a pregnancy but are not using any form of family planning[1]. Over the past two decades, reproductive health and family planning programs, seeking to reduce this unmet need for contraception, have increasingly included components to address women’s empowerment[2]. Yet virtually all the evidence comes from cross-sectional studies rather than interventions or longitudinal research. Women’s empowerment has been defined as the ability of the woman to formulate strategic choices and control resources and decisions that affect important life outcomes[3]. Women’s decision-making power, measured through the Demographic and Health Surveys (DHS), can be analyzed using a participation model which considers any form of women’s active role in decision-making (autonomous or joint decisions with the husband) versus all the decisions being made by the husband. Two-dozen DHS country reports demonstrate that contraceptive prevalence depends on the number of household decisions in which the woman participates[4–9]. Recent meta-analyses in 31 to 46 countries using DHS data confirmed the link between women’s participation in decision-making and their use of maternal health services[10] or family planning[11].

Age, education, and work for pay are the characteristics that most consistently determine women’s participation in household decision-making[12]. Yet, these variables are not factors that reproductive health and family planning programs can address directly through their services. Education represents a long-term investment, and affecting women’s employment would require the application of vast material resources. Specialized microcredit programs for women have produced mixed results[13–18]. Since the associations between directly measured women’s decision-making power and contraceptive use do not simply reflect the magnitude of the proxy variables of age, education and work[11, 19], it is theoretically possible to improve contraceptive use through initiatives that address women’s empowerment by other means. Do organizing services to reduce barriers for women, training providers to be aware of gender-based power dynamics, and involving men in counseling and services empower women? Furthermore, does increasing the availability of family planning information and services benefit women with lower levels of decision-making power to the same extent as it benefits women with higher levels of decision-making power? Generally, it is assumed that more empowered women would have the advantage in attaining access to family planning. Could a program’s efforts to widen access equalize access for women, regardless of their empowerment levels? Finally, is there an interaction between women’s decision-making power and literacy? Are women who have high levels of decision-making power but who are illiterate similarly affected by a family planning intervention as literate women who have lower levels of decision-making power?

This paper applies the participation model of women’s decision-making power in the analysis of data from a study of a family planning intervention in Jharkhand state in India to test if the intervention had any effect on women’s empowerment. Our intervention in Jharkhand, which expanded the availability of family planning information and methods and stressed the need for couple decision-making and communication, significantly reduced women’s unmet need for contraception in the intervention area compared to the control area[20]. The earlier analysis of the intervention data, however, did not consider women’s empowerment variables. This paper seeks to determine whether literacy moderated the effects on women’s decision-making power of the family planning intervention in Jharkhand and whether literacy and women’s decision-making power together moderated the effects of the intervention on met need for contraception. Met need is used because it more directly measures the ability of the woman to overcome barriers to access and thus is a more appropriate dependent variable for studies designed to test the effects of program efforts to increase access; contraceptive use confounds access to contraception with fertility desires and risk of pregnancy.

## Methods

The institutional review board of Georgetown University at Washington, DC approved all aspects of treatment of human subjects of the study design.

### Study setting

Unmet need for contraception was declining in India and it reached 13% in 2005–2006. Jharkhand, a state in Northeastern India, has been described as more patriarchal than Southern states in India[21]. In Jharkhand, according to the latest National Family Health Survey (NFHS-3), conducted in 2005–2006[22], only 37 percent of married women of reproductive age are literate and 60 percent are not exposed to any media. The NFHS-3 found that 35.7 percent of married women of reproductive age used contraception, including 28.2 percent in rural areas of the state. Female sterilization dominates the method mix, with 23.4 percent of Jharkhand’s married women of reproductive age using that method, followed by the pill (3.8%), the condom (2.7%), and scant use of other modern methods. Less than 5 percent of women use a traditional method. The method mix in rural areas mirrors that in the state. The public health system in these areas consists of subcenters reporting to a primary health center. In each sub-center, one auxiliary nurse midwife provides services to an average of five villages.

### Intervention

The family planning intervention that was designed to reduce women’s unmet need for contraception[11] was undertaken in three rural blocks in Jharkhand in 2004–2007. It was comprised of strengthening family planning generally and introducing a new family planning method, and was carried out in the Ormanjhi block, encompassing 89 villages and about 76,000 inhabitants. Burmu, encompassing 101 villages and about 77,000 inhabitants, served as a control site for the study. A third block that also received the intervention, Kanke, was excluded from the analysis in this paper, given its more urban structure than the other two blocks. Krishi Gram Vikas Kendra (KGVK), a nongovernmental organization (NGO) that works in Jharkhand and manages six subcenters in Ormanjhi, coordinated the intervention.

To strengthen family planning in the experimental villages, an NGO specializing in street theatre and puppet shows was hired to provide information about contraceptive methods, couple communication and decision making related to family planning and women’s reproductive rights. Public and private providers posted signs announcing that they offered various family planning methods. Wall paintings in public areas also informed village residents about the availability of a range of contraceptive methods, and providers conducted health fairs in villages. Providers were trained to offer family planning information and services which took into consideration gender power dynamics. No individual contraceptive method was stressed in the messages and information.

The second component of the intervention introduced a relatively new method, the Standard Days Method® (SDM), to public health centers run by the Ministry of Health and sub-centers run by NGOs. SDM is appropriate for women with cycles that usually range 26–32 days. It identifies days 8 to 19 of the menstrual cycle as the fertile window, i.e., the days when pregnancy is most likely. To prevent pregnancy, the couple avoids unprotected intercourse during the 12-day fertile window, which is identified by using a visual aid representing the menstrual cycle, a color-coded string of beads called Cyclebeads®. SDM efficacy rates, established in a clinical trial, are comparable to those of male condoms, the failure rate being less than 4 per 100 women years of correct use. Adoption of this method requires agreement by the couple rather than the wife alone. Providers were trained in SDM counseling; service delivery points were supplied with Cyclebeads®, the visual tool that supports correct use of SDM, and with simple leaflets presenting the method as a new family planning option in the context of informed choice. Anganwadi workers (community health workers) and village animators were also trained to offer information on family planning including SDM, and to provide SDM. The community health workers in particular were encouraged to reach out to men and couples. The intervention was not designed to address women’s empowerment broadly, but its emphasis on empowering women to know about and use family planning, and its promotion of couples’ joint decision-making concerning family planning use, were relevant to women’s empowerment in the intervention site. Furthermore, given that SDM is a couple method, and that it was being introduced as a new method of family planning in the intervention area, special care was taken to ensure that men were reached through the IEC [information, education and communication] efforts. In the control villages in Burmu, women had access to family planning through regular service channels; however, no special information was provided about availability of services nor was SDM offered in those villages.

### Data

Married women ages 15–49 who lived in these blocks had an equal opportunity to participate in the study. A research firm in New Dehli conducted the baseline and endline community surveys. The former was conducted in both blocks in late 2004-early 2005, three months before the intervention started in Ormanjhi, and the latter was conducted after the intervention had been in place for close to three years (2007). The pretest and posttest surveys were independent of each other. To minimize the number of randomly selected households lost due to addresses not found, the final sampling frame was obtained after thorough physical inspection of addresses. All married women of reproductive age were eligible to be interviewed within each selected household. Up to three repeat visits were undertaken early in the morning or late in the evening, to minimize the number of respondents not found at home.

Women who had no need for family planning, because they desired more children soon or were not at risk of pregnancy, were excluded from the analyses for this study, which focused directly on women with a need for contraception. Also, given the very small number of followers of religions other than Hinduism and Islam, only Muslim women were selected for comparison with Hindu women, who were the majority in the sample.

### Variables

- Women’s Decision-making Power. In this study, women’s decision-making power was measured though normative beliefs concerning whether women should be involved in various household decisions. The questionnaire included a 5-item question on household decision-making, consistent with questions in the DHS: “In a couple, who do you think should have the greater say in each of the following decisions: the husband, the wife, or both equally?” The decisions were: making large household purchases; making small daily household purchases; deciding what to do with the money she earns for her work; deciding when to visit family, friends, or relatives; and deciding how many children to have and when to have them. The few cases choosing the response options “Don’t know/depends” were excluded from analysis. Implementing the participation model in the measurement of normative beliefs, 1 point was assigned to Wife and Both equally, and 0 points were assigned to Husband only.
- Need for Contraception. To determine eligibility for inclusion in the analysis, a score of 1 was assigned to: (a) pregnant women who had wanted their last child later or had not wanted more children at all, (b) nonpregnant women who were using any family planning method, and (c) nonpregnant women who were not using family planning despite their not wanting a child in the next two years and being at risk of pregnancy (or did not respond whether she wanted the child). A score of 0 was assigned if (a) a pregnant woman said that the child was intended, or (b) a nonpregnant woman was not using family planning because she wanted to have children in the next two years or was not at risk of pregnancy (infecund, menopausal, postpartum amenorrhea, not sexually active).
- Met Need*.* A score of 1 was assigned to the woman in need of contraception if she said that she was using any *modern* family planning method and a 0 if she was pregnant, was using a traditional method, or was using no method.
- Age. The woman’s age was calculated considering her birth date and the date of the interview. Two questions were asked: “In which month and year were you born?” and “What age did you reach in your last birthday?” Inconsistencies were corrected where possible.
- Children. Women were asked how many living children they had.
- Literacy*.* Women were asked, “Have you ever gone to school?” and, “What was the highest year of studies you attained?” Women who responded “Primary instruction” or less were given a card with a sentence and asked to read it. To translate educational attainment into a single score, the two variables were combined and produced the following scale: 0 = unable to read, whether the woman had formal education or not (70.8%) and 1 = reads part or all the sentence and/or has primary, secondary, or higher education (29.2%).
- Work. The woman was asked whether she worked at the time of the interview and whether she had worked in the past 12 months. (“As you know, some women work for a pay in cash or in kind. Others sell things, have small businesses, or work family land or in family enterprises. Are you currently doing any of these jobs? Have you worked in the past 12 months?”) Her responses were coded 0 = did not work and 1 = worked and/or is working now, regardless of whether she received payment or not, and the type of payment.
- Religion. Women were asked whether they were Hindu, Muslim, Christian, Sikh, Jain, or Buddhist. Muslims received a score of 0, Hindus a score of 1, and the others a missing value.
- Listening to Radio. Women were asked if they listened to radio and frequency of radio listening: never (0), less than once a week (1), at least once a week (2), or almost every day (3).
- Watching Television. Women were asked if they watched television and frequency of television watching: never (0), less than once a week (1), at least once a week (2), or almost every day (3).
- Visited Health Center*.* Women who had visited a health center in the past 12 months were given a score of 1. Those who did not received a 0.
- Received Visit. Similar scores were assigned to women who in the past 12 months had received the visit of anyone who talked about family planning.

### Analyses

Chi-square (χ^2^) and the *t*-test for independent samples were employed to analyze simple pre-post data, and the following generalized linear model evaluated the effects of the intervention on women’s decision-making power:

1 $$\lambda =\beta_{0}+\beta_{i}\left(\delta \pi \right)+\left[\beta_{j}\xi_{k}\right]+\epsilon$$

where λ is the decision-making power measure and β and ϵ are the regression coefficients and the error term. The main target of the analysis was the δ x π interactive effect on decision-making power, where δ is a fixed-effects treatment factor defined as 1 = Burmu pretest, 2 = Ormanjhi pretest, 3 = Burmu posttest, and 4 = Ormanjhi posttest and π is a fixed-effects literacy factor with two levels (literate, illiterate). The other component of Equation 1, ξ_*k*_, is a set of main-effect covariates that includes age, religion, children, radio, TV, and work. A similar model, but with the focus on the 3-way treatment x literacy x decision-making power interaction and having met need for contraception as the dependent variable (γ) was employed to assess the moderating roles of women’s literacy and decision-making power in the intervention process:

2 $$\gamma =\beta_{0}+\beta_{i}\left(\delta \pi \lambda \right)\;+\;\left[\beta_{j}\xi_{k}\right]\;+\epsilon$$

## Results

**Figure 1:**
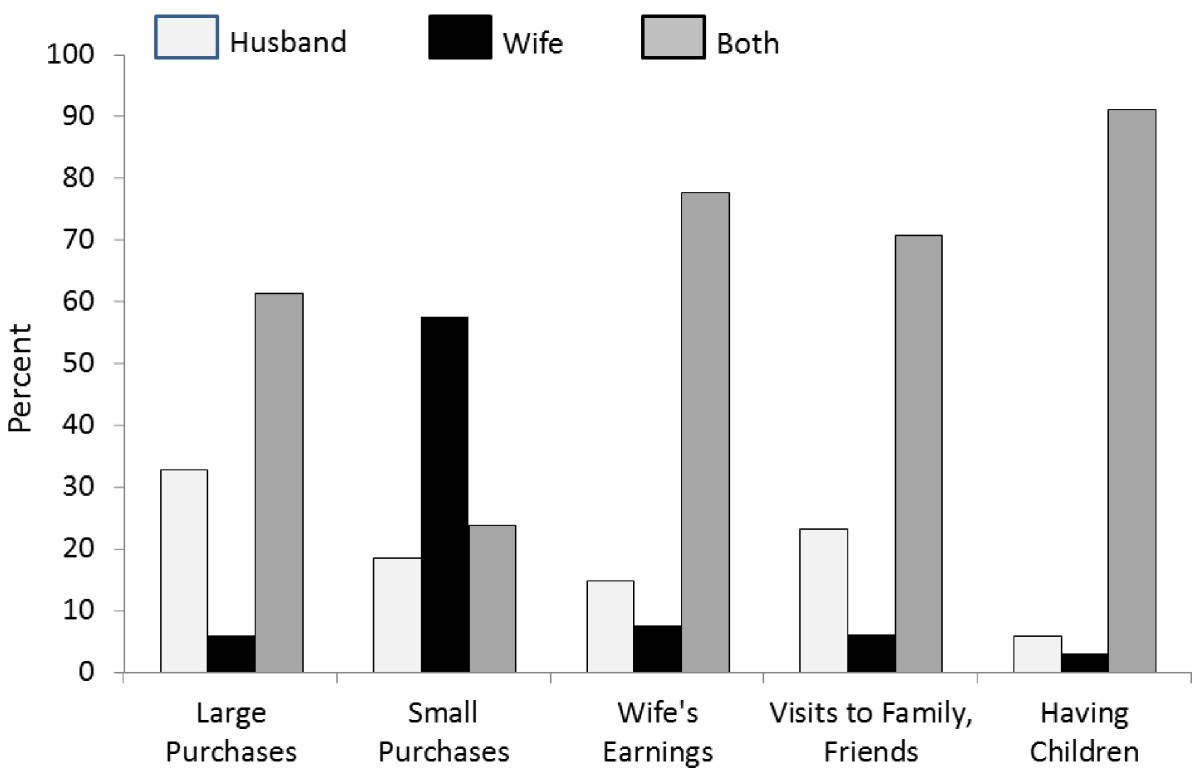
**Women’s normative beliefs concerning who should do what at home, by decision area.**

Table 1 presents descriptive statistics which reveal a moderately high level of met need in rural Jharkhand as well as an increase from pretest to posttest in the intervention area, Ormanjhi, but not in Burmu, the control area. Regarding the covariates, the results show that literacy, watching TV, proportion of Muslims, and family planning visits received, all increased from pretest to posttest in both groups and thus these changes can be regarded as parts of maturation processes in rural Jharkhand. The proportion of women working increased in the control but not in the intervention site; since the difference between blocks at the pretest was significant (χ^2^ = 7.564, *p* < .000), this dynamic may be understood as the women from Burmu catching up with their neighbors. The fact that the proportion of Muslims was greater in Burmu at the pretest (χ^2^ = 10.319, *p* < .000) may be relevant in this respect, considering their general employment level (35% working, versus 57% among Hindus). Visits to health centers significantly decreased in Ormanjhi but not in Burmu. The results shown in Figure 1, which refer to the combined intervention and control samples, suggest that a practical, egalitarian model of domestic decision-making prevails in rural Jharkhand: while making decisions about small purchases appears to be within women’s autonomous decision-making purview, the other four are ones in which joint decisions with the husband are expected.

**Table 1:** **Pretest and posttest means for study variables among women with a need for contraception and test for differences between phases, per site**

Variable	Study sites					
	Burmu (control)			Ormanjhi (intervention)		
	Pretest	Posttest	Difference	Pretest	Posttest	Difference
Met need for contraception	0.642	0.684	χ^2^ = 1.890	0.606	0.703	χ^2^ = 8.808**
Age of informant	30.94	30.50	*t* = 0.863	30.03	30.69	*t* = 1.216
Her number of children	2.921	3.110	*t* = 1.760	2.787	2.934	*t* =1.266
Her literacy	0.208	0.379	χ^2^ = 33.770***	0.232	.373	χ^2^ = 20.489***
Frequency of hearing radio	1.226	1.229	*t* = 0.036	1.081	1.216	*t* = 1.580
Frequency of watching TV	0.869	1.341	*t* = 5.657***	0.928	1.812	*t* = 10.266***
Whether infoermant works	0.440	0.628	χ^2^ = 33.658***	0.528	0.574	χ^2^ = 1.814
Whether Hinduism is professed	0.935	0.848	χ^2^ = 18.937***	0.875	0.800	χ^2^ = 8.977**
Wether informant was visited	0.091	0.182	χ^2^ = 16.773***	0.128	0.272	χ^2^ = 28.469***
Whether informant visited HC	0.398	0.339	χ^2^ = 3.591	0.494	0.376	χ^2^ = 12.109***
(N)	(505)	(446)		(470)	(394)	

*p < .05, **p < .01, ***p < .001, two-tailed.

**Figure 2:**
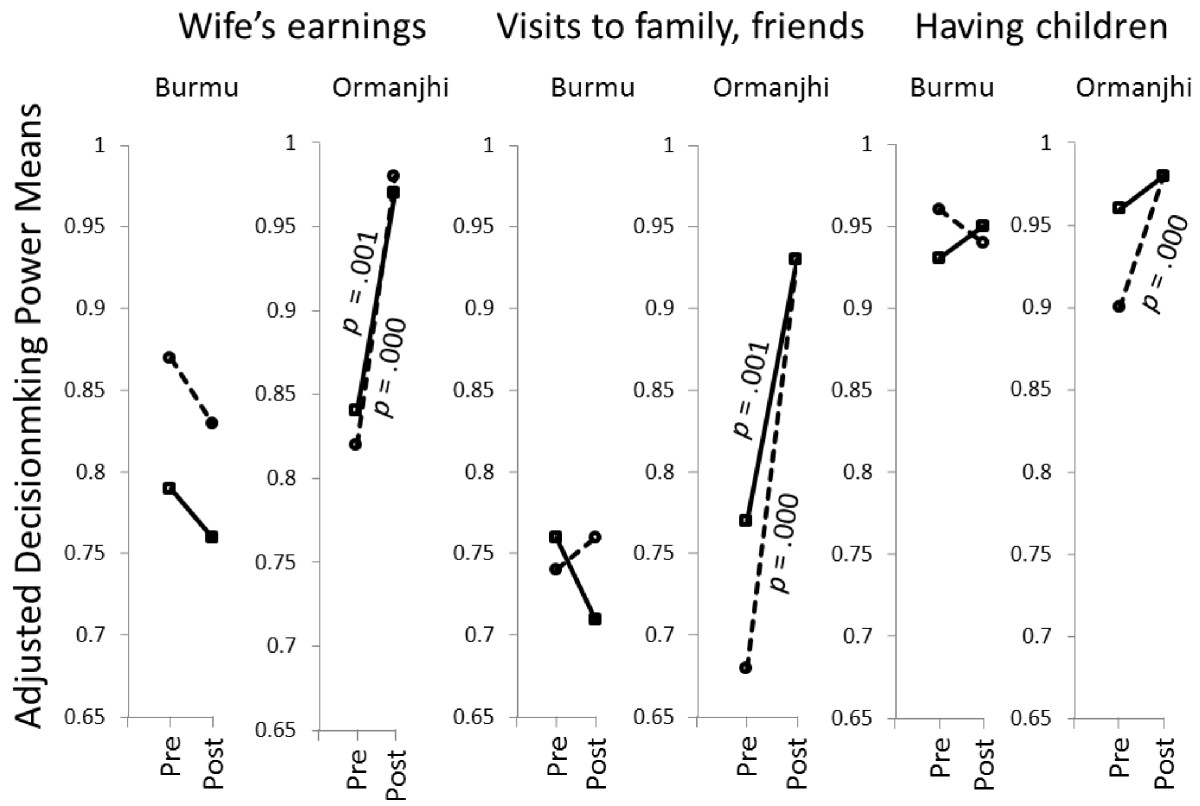
**Adjusted power means for literate (solid lines) and illiterate women (dotted lines) at pretest and posttest, per site and decision area, and significant changes (** ***p*** **< .05).**

Table 2 and Figure 2 present results from generalized linear models which had women’s decision-making power scores as dependent variables. Neither decision-making power to make large purchases nor to make small purchases was affected by the intervention. Regarding the role of literacy as a moderating variable, the pairwise comparisons indicate that in the intervention area, women’s power to decide on having children increased among illiterate women but not among literate women. The intervention was associated with increased decision-making power for women regarding use of wife’s earnings and making visits – for both illiterate and literate women. Regarding the other covariates, younger women held normative beliefs suggestive of greater power in decisions on having children; number of living children positively influenced decision-making power for small purchases and disposal of earnings; women who listened to radio never or infrequently tended to have more decision-making power regarding making visits; women who worked were more likely to say that they should participate in decisions on large purchases for the home and regarding disposal of earnings; and those who were visited by a community health worker to talk about family planning considered themselves more empowered to make decisions on visiting family and friends or having children.

**Table 2:** **Odds ratios from generalized linear models predicting women’s decision-making power from treatment x literacy interaction and covariates, per decision-making item**

Treatment	Literacy	Large purchases	Small purchases	Earnings	Visits	Having children
Ormanjhi posttest	Literate	1.000 (Ref.)	1.000 (Ref.)	1.000 (Ref.)	1.000 (Ref.)	1.000 (Ref.)
	Illiterate	0.809	0.866	1.222	1.125	1.303
Burmu posttest	Literate	0.735	0.485*	0.096***	0.198***	0.506
	Illiterate	0.794	0.712	0.140***	0.257***	0.398
Ormanjhi pretest	Literate	1.258	0.915	0.160***	0.275***	0.533
	Illiterate	0.808	0.691	0.138***	0.172***	0.228**
Burmu pretest	Literate	1.099	0.784	0.110***	0.257***	0.343
	Illiterate	0.743	0.746	0.201**	0.228***	0.539
Age		1.013	1.010	0.987	1.000	0.971*
Religion		1.326	1.140	1.263	1.153	1.372
Children		1.027	1.114*	1.168**	1.030	1.147
Radio		0.925	0.965	0.970	0.817***	1.005
TV		0.989	0.990	0.931	1.030	0.909
Work		1.234*	1.258	1.473**	0.980	0.900
Was visited		1.050	1.333	1.143	1.416*	3.440***
Visited HC		0.966	0.939	1.303	1.066	1.040
Wald chi-square for treatment x literacy interaction		7.247	9.409	46.243***	62.345***	20.888**
(N)		(1,790)	(1,788)	(1,677)	(1,789)	(1,782)

*p < .05, **p < .01, ***p < .001.

Nonresponse was greater for the question on use of wife’s earning than for the other questions, because women who did not work for pay tended to choose “no response” in answer to this question. To avoid a loss of cases, a sum of women’s decision-making power scores was computed without the question on disposition of women’s earnings. With a total number of four items dichotomously scored as 0 or 1, the summed score could range from 0 through 4. The internal-consistency reliability of the sum of the four items’ scores was not fully satisfactory (illiterate α = .58; literate α = .57). That is, women who were powerful in one area were not necessarily powerful in a different area. That the decision-making power scale αs were weaker than reported in an analysis at the national level for India can be explained by the fact that the current analysis referred to normative beliefs and used the participation model of decision-making while the study at the national level entailed self-reported behavior using a control model of decision-making which defines a hierarchy of women’s self-perceived decision-making power that ranges from lack of participation to joint decisions with the husband to women’s autonomous decision-making[11]. For the analysis of the function of women’s decision-making power in the intervention to increase met need for contraception, the sum was dichotomized at the median, yielding a group of women with relatively less decision-making power (47.4% with a summed score from 0 through 3) and a group with relatively more decision-making power (52.6% with a score of 4).

**Figure 3:**
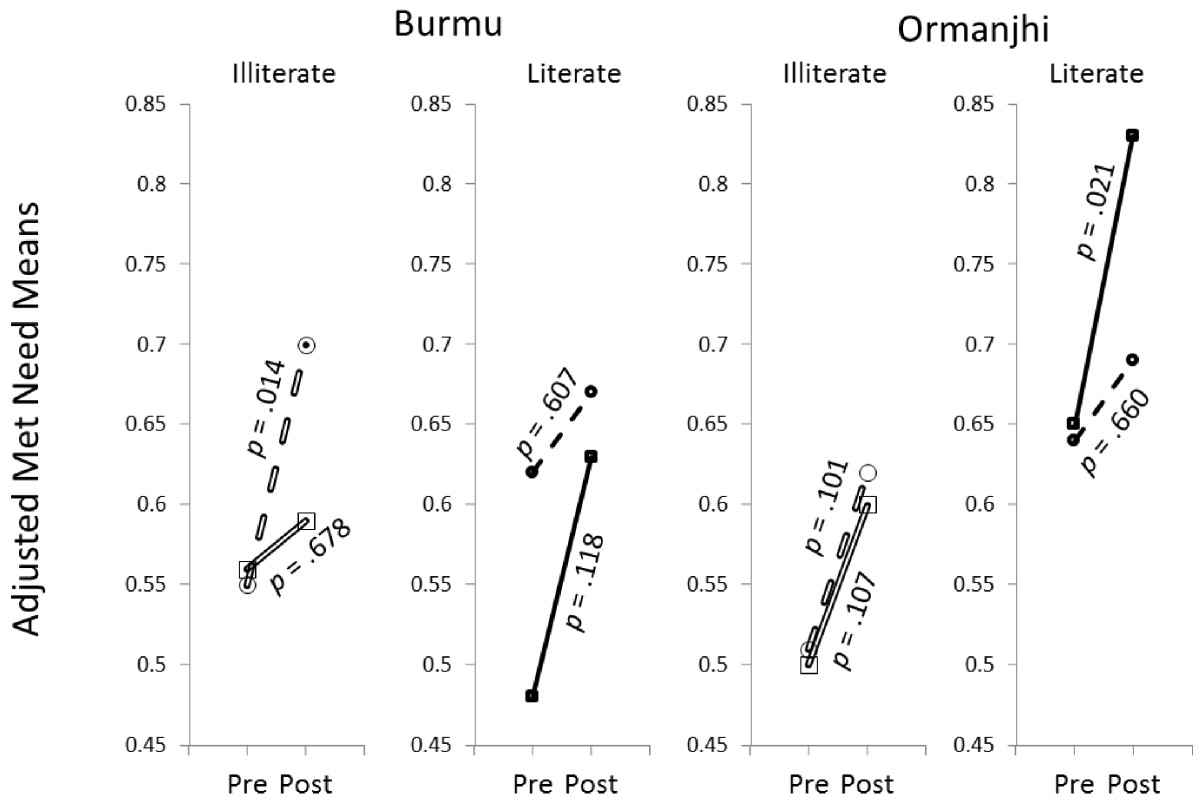
**Adjusted met need means for women with relatively more power (solid lines) and women with relatively less power (dotted lines) at pretest and posttest, per site and literacy level, and significance of changes (** ***p*** **< .05).**

Table 3 and Figure 3 present results from the generalized linear model with met need as the dependent variable. A maturation effect is suggested by the adjusted means: the eight subgroups (intervention and control groups at pre- and posttest and by literacy level) presented increased met need levels from pretest to posttest. However, the rates of change differed. It is evident that the intervention did not improve met need among women with relatively less decision-making power who were illiterate, but the change for women with relatively more power who were illiterate was greater in the intervention area Ormanjhi, than in the control area Burmu. Similarly, the steepness of the curves for literate women with relatively less decision-making power was virtually the same at the two sites, while it was steeper in the intervention area than the control area for literate women with relatively more decision-making power. Thus, the findings strongly suggest that, regardless of whether the woman was literate or illiterate, she was more likely to have her need for family planning met at the posttest in the intervention than the control site if she had relatively more decision-making power than if she had relatively less decision-making power. As for differences observed between sites at the baseline, they had opposite signs depending on whether the women were literate or illiterate. It can be seen in Figure 3 that Burmu (control) presented slightly greater levels of met need than Ormanjhi (intervention) among illiterate women at the pretest, while the opposite is clearly visible among literate women. (Since there were more illiterate women in the total sample, Burmu presented a no significant greater met need than Ormanjhi at the pretest in Table 1.) Concerning the covariates, religion had the strongest effect on met need. Hindus were nearly five times as likely as Muslims to have a met need. Older women who had more children and were frequent TV viewers were more likely to have a met need than younger women who had fewer children and were less frequent TV viewers.

**Table 3:** **Odds ratios from generalized linear model predicting met need for contraception from treatment x literacy x power interaction and covariates**

Treatment	Literacy	Power	Met need	N
Ormanjhi posttest	Literate	4 points	1.000 (Ref.)	79
		0-3 points	0.451*	63
	Illiterate	4 points	0.305***	141
		0-3 points	0.337**	103
Burmu posttest	Literate	4 points	0.348**	75
		0-3 points	0.428*	91
	Illiterate	4 points	0.294***	143
		0-3 points	0.474*	127
Ormanjhi pretest	Literate	4 points	0.393*	62
		0-3 points	0.370*	44
	Illiterate	4 points	0.204***	169
		0-3 points	0.218***	174
Burmu pretest	Literate	4 points	0.193***	53
		0-3 points	0.345*	46
	Illiterate	4 points	0.266***	201
		0-3 points	0.250***	184
Age			1.061***	1755
Religion			4.750***	1755
Children			1.290***	1755
Radio			1.083	1755
TV			1.113*	1755
Work			1.127	1755
Was visited			1.247	1755
Visited a HC			1.007	1755
Wald chi-square for treatment x religion interaction			39.361***	

*p < .05, **p < .01, ***p < .001.

## Discussion

That women of the intervention site were more empowered in terms of decision-making over the three year period of the study than those of the control site is well established by the research results: the differences in normative beliefs about wife’s participation in decision-making between women in Burmu (control) and Ormanjhi (intervention) at the baseline were all non-significant, while significant pretest-posttest changes only occurred in the intervention site, Ormanjhi. These findings suggest that the intervention had an effect on women’s power in decision-making and that alternative interpretations related to selection bias, local history, maturation, regression towards the mean, attrition, and other threats to the internal validity of quasi-experimental designs[23] can be reasonably ruled out. It is generally accepted that a nonequivalent control group quasi-experiment with pretest and posttest approximates answers from randomized experiments[24], especially if it tackles the main limitations of the research design: selection bias and attrition[25]. In this study, attrition was avoided through independent sampling of respondents at pretest and posttest. Selection bias was ameliorated by having a comparison group living close to the intervention group. Burmu and Ormanjhi are contiguous blocks within the district of Ranchi in the state of Jharkhand that share the same ecological, socioeconomic and cultural configurations. Their comparability was made evident by the finding of similar pretest-posttest changes in literacy, watching TV, proportion of Muslims, and visits received, although the observed increase in women working in Burmu but not in Ormanjhi and the differences in the proportion of Muslims and women working at the baseline suggest that some historical processes may have differentiated the groups.

The observed changes, however, did not involve all the decision areas and sub-groups of women. Both literate and illiterate women of the intervention site increased their levels of decision-making power with respect to disposition of wife’s earnings and visits to relatives and friends but not regarding large or small purchases. A possible explanation for this finding is that the nature of the intervention affected dimensions of women’s empowerment differently. It is likely that the messages used in the intervention were more relevant to women’s decision-making about their own earnings and about freedom of movement, namely to visit family and friends, than about specific material possessions, namely making purchases. A revealing finding of the study in this respect is that decision-making power regarding making visits and having children was significantly greater among women who had received family planning visits than those who had not. The finding that literate women, whose normative beliefs entailing earnings and visits were changed by the intervention, did not seem to have their beliefs changed about their decisions on having children, can be explained by a ceiling effect. Whereas normative beliefs regarding fertility decision-making had considerable room for change among illiterate women in Ormanjhi, this was not the case for literate women.

Regarding the specific causes of the observed changes in women’s decision-making power, it seems unlikely that their interaction with providers at service delivery points in the intervention group was the only cause of the observed empowerment. The women who visited a health center during the period of the study did not present greater decision-making power levels than those who did not. But the decision-making power levels of those who received a visit of someone who talked about family planning did increase, which suggests a direct influence of the intervention’s outreach efforts. Normative beliefs concerning visits to family and friends and having children were likely changed by the visits received at home. Outreach workers were trained to emphasize the convenience of traveling to a health center or sub-center and to discuss decision-making about fertility with women and men, which would explain why decision-making power changes were observed in these decision areas and not those related to purchases.

It is not possible to determine whether the influence of the intervention was limited to the 27% of women in the intervention site who were visited during the study period. It is also possible that the women who were visited in turn influenced some of their neighbors. If this was the case, the stable unit treatment value assumption of the Rubin model, which establishes that the treatment obtained by one individual has no effect on the set of potential outcomes of any other individual, was violated[26, 27]. Nonetheless, while it is very likely that some women in Ormanjhi influenced by the intervention in turn influenced others, the likelihood that women in Ormanjhi affected the outcomes of women in Burmu is much less likely. As for the street theatre and puppet shows, these may have also contributed to the observed empowerment.

Since outreach actions are frequently implemented by family planning programs, an important practical question refers to the extent to which such actions by themselves, without community-level media (in the case of this intervention, street theatre and puppet shows), are able to produce the effective empowerment seen in this analysis. Despite these questions, the research showed that women’s empowerment in several household decision-making areas can be enhanced by family planning interventions.

Whether the empowerment contributed to meeting the needs of the women for contraception is more difficult to establish because all the subgroups of women at both control and intervention sites underwent positive changes regarding met need for contraception, which suggests an underlying shift in Jharkhand society. Compelling evidence suggesting a causal link with empowerment is the observation that the woman was more likely to exhibit an enhanced met need at the posttest in the intervention than the control site if she was literate and had relatively more power in decision-making, than if she was literate and had relatively less power in decision-making. The intervention might have enhanced met need by moving some literate women from the relatively less decision-making power to the relatively more decision-making power category.

A typical ethical limitation of the study was the assignment of one of blocks to the control condition, which implied that Burmu women lacked the opportunity to receive the enriched information and services received by Ormanjhi women. The state of Jharkhand, however, was expected to scale-up the intervention.

## Conclusions

Theoretical, methodological, and practical lessons can be drawn from this study. First, whereas most research has emphasized the cultural and social determinants of women’s empowerment, the analysis in this paper demonstrates that women’s empowerment not only is determined by such macro social forces but can be influenced by family planning programs that include attention to gender dynamics. While the intervention in Jharkhand did not have stated women’s empowerment objectives, it did include attention to gender dynamics, and its materials and messages incorporated gender transformative messages related to family planning use.

The specific paths through which programs can empower women and affect contraceptive use that were discovered in this study suggest that outreach efforts by themselves are not enough to empower women and increase contraceptive use. Visits received by women also increased in Burmu. The Burmu outreach workers, however, were not encouraged to stress the gender issues that were addressed in Ormanjhi.

It is of course important to note that these findings pertain to Northern India in areas of a state which already had a moderately high level of met need for contraception when the intervention started. Whether similar results are found in different contexts, including in Sub-Saharan Africa where contraceptive use generally remains low, and the links between empowerment and family planning may be more tenuous[28], only can be established by replications of the study outside India. Still, the findings of this analysis support the hypothesis that family planning programs that address gender are contributing to the empowerment of women.

## Authors’ original submitted files for images

Below are the links to the authors’ original submitted files for images. Authors’ original file for figure 1 Authors’ original file for figure 2 Authors’ original file for figure 3
