# Supplementary figures and images for: Increasing literate and illiterate women’s met need for contraception via empowerment: a quasi-experiment in rural India
Source: Reprod Health. 2014 Oct 21;11:74. doi: 10.1186/1742-4755-11-74 (PMC4221697; doi:10.1186/1742-4755-11-74)

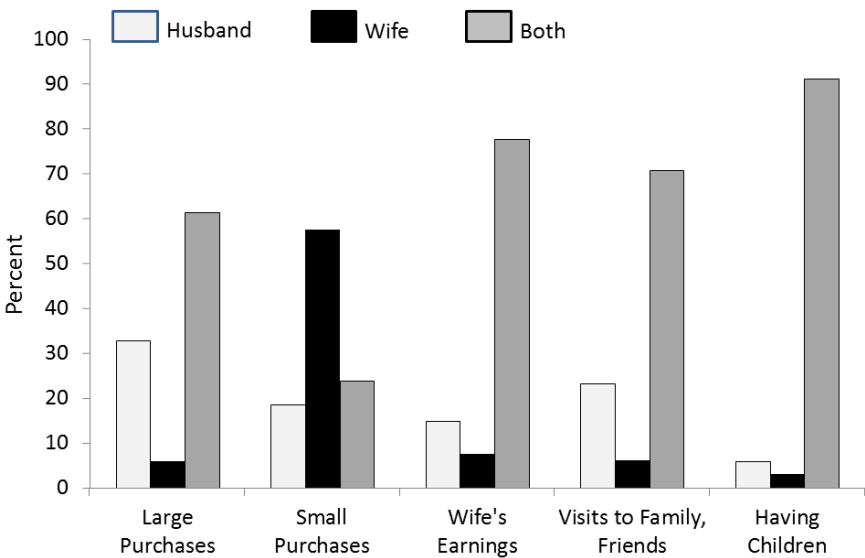

Supplement: Supplementary file 1 — Authors’ original file for figure 1 [file 12978_2013_325_MOESM1_ESM.pdf]

Adjusted Met Need Means

Burmu

Ormanjhi

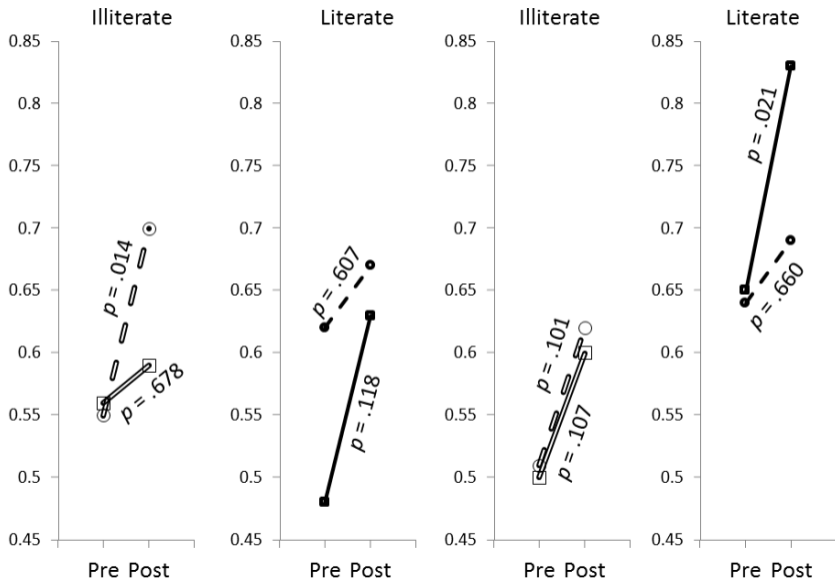

Supplement: Supplementary file 3 — Authors’ original file for figure 3 [file 12978_2013_325_MOESM3_ESM.pdf]
