# Supplementary material for: Increasing literate and illiterate women’s met need for contraception via empowerment: a quasi-experiment in rural India
Source: Reprod Health. 2014 Oct 21;11:74. doi: 10.1186/1742-4755-11-74 (PMC4221697; doi:10.1186/1742-4755-11-74)
Supplement: Supplementary file 2 — Authors’ original file for figure 2 [file 12978_2013_325_MOESM2_ESM.pdf]

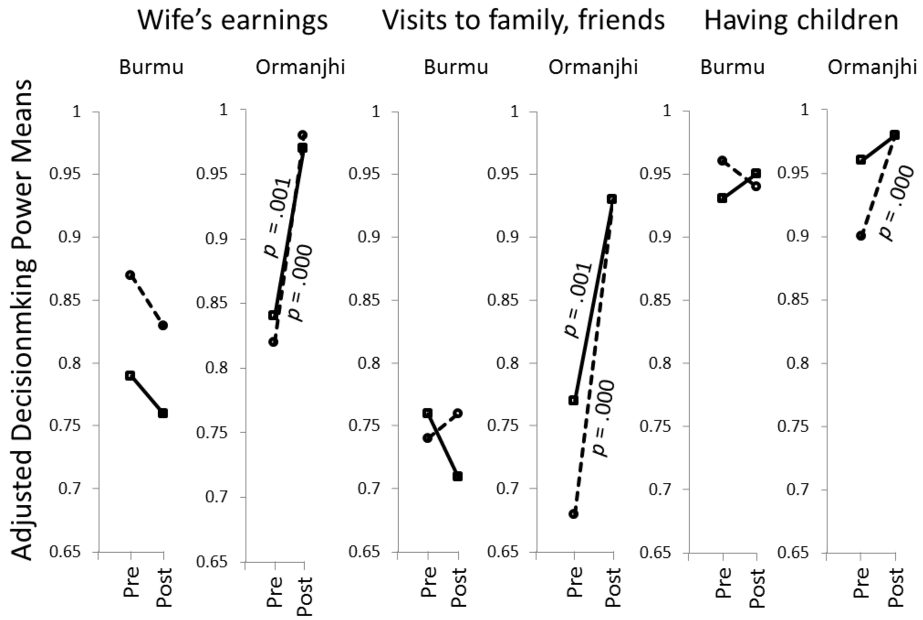
Wife's earnings
Visits to family, friends
Having children
Burmu
Ormanjhi
Burmu
Ormanjhi
Burmu
Ormanjhi
Adjusted Decisionmking Power Means
1
0.95
0.9
0.85
0.8
0.75
0.7
0.65
Pre
Post
p = .001
p = .000
p = .001
p = .000
p = .000
